# Lipopolysaccharide-Induced Nitric Oxide and Prostaglandin E2 Production Is Inhibited by Tellimagrandin II in Mouse and Human Macrophages

**DOI:** 10.3390/life11050411

**Published:** 2021-04-30

**Authors:** Chun-Yu Lin, Shih-Han Kao, Ling-Chien Hung, Hsin-Ju Chien, Wen-Hung Wang, Yu-Wei Chang, Yen-Hsu Chen

**Affiliations:** 1 Division of Infectious Diseases, Department of Internal Medicine, Kaohsiung Medical University Hospital, Kaohsiung Medical University, Kaohsiung 807, Taiwan; infectionman@gmail.com (C.-Y.L.); flutetina@gmail.com (H.-J.C.); bole0918@gmail.com (W.-H.W.); 2 School of Medicine, Graduate Institute of Medicine, College of Medicine, Center for Tropical Medicine and Infectious Diseases Research, Kaohsiung Medical University, Kaohsiung 807, Taiwan; potentia@gate.sinica.edu.tw (S.-H.K.); lavender99kimo@yahoo.com.tw (L.-C.H.); golden3p@gmail.com (Y.-W.C.); 3 Department of Surgical Sciences, Uppsala University, 751 23 Uppsala, Sweden; 4 Department of Medical Biochemistry and Microbiology, Uppsala University, 751 23 Uppsala, Sweden; 5 Department of Biological Science and Technology, College of Biological Science and Technology, National Chiao Tung University, Hsinchu 300, Taiwan

**Keywords:** Tellimagrandin II, macrophage, inflammation, nitric oxide, cyclooxygenase-2

## Abstract

Sepsis develops from a serious microbial infection that causes the immune system to go into overdrive. The major microorganisms that induce sepsis are Gram-negative bacteria with lipopolysaccharide (LPS) in their cell walls. Nitric oxide (NO) and cyclooxygenase-2 (COX-2) are the key factors involved in the LPS-induced pro-inflammatory process. This study aimed to evaluate the effects of polyphenol Tellimagrandin II (TGII) on anti-inflammatory activity and its underlying basic mechanism in murine macrophage cell line RAW 264.7 and human monocyte-derived macrophages. Macrophages with more than 90% cell viability were found in the cytotoxicity assay under 50 μM TGII. Pre- or post-treatment with TGII significantly reduced LPS-induced inducible nitric oxide synthase (NOS2) protein and mRNA expression, reducing LPS-induced COX-2 protein. Downstream of NOS2 and COX-2, NO and prostaglandin E2 (PGE2) were significantly inhibited by TGII. Upstream of NOS2 and COX-2, phospho-p65, c-fos and phospho-c-jun were also reduced after pre-treatment with TGII. Mitogen-activated protein kinases (MAPKs) are also critical to nuclear factor kappa-light-chain-enhancer of activated B cells (NF-κB) stimulation, and phospho-p38 expression was found to have been blocked by TGII. TGII efficiently reduces LPS-induced NO production and its upstream regulatory factors, suggesting that TGII may be a potential therapeutic agent for sepsis and other inflammatory diseases.

## 1. Introduction

Sepsis is a lethal host response to microbial infection, and its high mortality is related to the imbalance of inflammatory mediators. As Gram-negative bacteria cause severe infection [1,2], sepsis can lead to multiple organ dysfunction, including arrhythmias and heart failure [3,4]. Although great progress has been made in the understanding and treatment of sepsis and its complications, the sepsis-related mortality rate has not improved [2]. In a retrospective cohort study of sepsis in an emergency department, sepsis-related death occurred in 61 out of 98 cases (62%) [2]. Improving sepsis outcomes with new treatments remains challenging.

Lipopolysaccharide (LPS) is a major component of the outer membrane of Gram-negative bacteria and is known to be a key pathogenic stimulator for multiple organ dysfunction. In the case of sepsis, circulating LPS as a pathogen-associated molecular pattern (PAMP) stimulates the innate immune system and mediates local or systemic inflammation [5,6,7]. Macrophages play a critical role in innate immunity and serve as the first line of defense against pathogen infection. Microbial components are the potential stimulators for macrophage activation. Sepsis is associated with the imbalance between pro-inflammatory and anti-inflammatory factors induced by macrophages [8]. Patients with sepsis show a higher inflammatory response, including cytokine secretion, nitric oxide (NO) production and prostaglandin synthesis [9]. Additional study has revealed that prostaglandin E2 (PGE2) is mainly synthesized by cyclooxygenase-2 (COX-2), which also leads to inflammatory symptoms and signs related to sepsis [10]. COX-2 can be overexpressed after LPS stimulation [11]. Strikingly, two recently published studies showed that itaconate markedly suppresses the production of proinflammatory mediators in LPS-stimulated macrophages and reduces sepsis and psoriasis, providing a new explanation for the switching of macrophages from a pro-inflammatory status to an anti-inflammatory state, thereby limiting potential damage and promoting tissue repair under proinflammatory conditions [12,13]. This explanation provides a rationale for future investigation and therapeutic intervention.

Tellimagrandin II (TGII), as a plant polyphenol extracted from the shells of *Trapa bispinosa* [14] or the petals of *Filipendula ulmaria* [15], is a natural product with broad medicinal use worldwide. Polyphenols are able to serve as secondary metabolites and play a vital defense against external stress such as pathogenic infection and ultraviolet radiation [16], and have been shown to have antioxidant, anti-inflammatory, anticancer, and antimicrobial effects [17,18,19,20]. In addition, due to the low cytotoxicity of these compounds, researchers have shown a keen interest in the potential health benefits of natural polyphenols and the development of products made from fruits or foods with substantial polyphenol content to explore additional applications in medicinal therapies and human health [21,22,23,24].

Our previous study reported that TGII inhibits the antibiotic resistance of drug-resistant *Staphylococcus aureus* [25]. In addition, TGII is a promising source for novel inhibitors of histidine decarboxylase (HDC) [15]. However, it remains unclear whether or in what manner TGII influences inflammation. Therefore, the present study aimed to evaluate anti-inflammatory activity induced by TGII and its specific effects on LPS-induced NO and PGE2 production.

## 2. Materials and Methods

### 2.1. Reagents and Antibodies

TGII was identified and provided by Dr. Lih-Geeng Chen (National Chiayi University, Chiayi, Taiwan) [25], while LPS (Escherichia coli O26:B6, L2654) was purchased from Sigma (St. Louis, MO, USA). Nitric oxide synthase 2 (NOS2) antibody was purchased from BD Biosciences (San Jose, CA, USA). The primary antibodies, including anti-phospho-p38 (Thr180/Tyr182), anti-phospho-ERK1/2 (Thr202/Tyr204), anti-phospho-SAPK/JNK (Thr183/Tyr185), anti-phospho-IκB-α (Ser32/36), anti-p38, anti-ERK1/2, anti-SAPK/JNK, anti-COX-2, anti-c-Fos (9F6), anti-phosphor-c-Jun (Ser73), and anti-phospho-NF-κB p65, were purchased from Cell Signaling Technology (Denver, MA, USA). The primary antibodies, including anti-lamin B (Santa Cruz Biotechnology, Dallas, TX, USA), anti-β-tubulin (Abcam, Cambridge, MA, USA) and anti-GAPDH (Abcam, Cambridge, MA, USA), were also used in this study.

### 2.2. Cell Culture

RAW 264.7 cells, murine macrophages, were purchased from the Bioresource Collection and Research Center (Hsinchu, Taiwan). RAW 264.7 cells were cultured in Dulbecco’s Modified Eagle Medium (DMEM) (GIBCO, Carlsbad, CA, USA) containing 10% fetal bovine serum (FBS) (Biological Industries, Cromwell, CT, USA), 100 μg/mL streptomycin, 100 U/mL penicillin, 25 μg/mL amphotericin B (Biological Industries, Cromwell, CT, USA), and 2 mM l-glutamine in a 37 °C incubator with 5% CO_2_.

### 2.3. Cell Viability

TGII-stimulated cytotoxicity in macrophages was assessed using Alamar Blue Cell viability kit (Serotec Ltd. Scandinavia, Hamar, Norway). In a 96-well plate, 10% Alamar Blue was added to 2 × 10^4^ cells (200 μL/well), which were then treated with medium, vehicle (0.1% DMSO), or TGII (10, 25, 50 μM) for 24 h. Colorimetric analyses were measured by an ELISA plate reader at 570 and 600 nm, and the cell viability in TGII-treated cells was calculated relative to that of controls.

### 2.4. Measurement of NO Production

The nitrite, a stable metabolite of NO, was an indicator of NO production [26] and detected using the Griess reagent system (Promega Biotech Co., Ltd., Madison, WI, USA) according to the manufacturer’s instructions. The 50 μL supernatants were transferred into a 96-well plate, and then we added 50 µL of Griess reagent. In the Griess reagent system, nitrite standard, 0.1 M sodium nitrite, was prepared for standard curve from the concentration 100 μM to 1.56 µM. After color development for 10 min, the absorbance at a wavelength of 520 nm was measured, which was calculated as the nitrite concentration using the standard curve.

### 2.5. Preparation of Nuclear and Cytosolic Fractions

The fractions of cytosolic and nuclear extracts were isolated from the cells according to a previously described protocol [27]. After LPS stimulation for 10, 15, 30, and 60 min, the cytosolic and nuclear extracts were harvested by the previously described method [28].

### 2.6. RT-PCR and Western Blot Analysis

The drug-treated cells (the treatment period was specified experiment by experiment in the figure legends) were collected, and we performed the reverse transcription-polymerase chain reaction (RT-PCR) analysis [27]. For RT-PCR, the mRNA was extracted using the RNA spin Mini RNA Isolation Kit (GE Healthcare, Buckinghamshire, UK), and then was performed by a Roche Light Cycler (Mannheim, Germany). Primers of RT-PCR are described in Supplementary Table S1. In addition, the drug-treated cells were collected to analyze the protein expression by Western blot analysis [27]. GAPDH was an internal control for RT-PCR.

### 2.7. Enzyme-Linked Immunosorbent Assay (ELISA)

RAW 264.7 cells (4 × 10^5^ cells/well) were seeded in 12-well plates. After the incubation with TGII and LPS for 6 h, the supernatants were collected and measured for PGE2 proteins by ELISA kits (R&D Systems, Minneapolis, MN, USA) according to the manufacturer’s instructions. 

### 2.8. Isolation of Human CD14^+^ Monocytes and Stimulation into Macrophages

This experiment was approved by the Institutional Review Board of Kaohsiung Medical University Hospital (KMUH-IRB-20140303). CD14^+^ monocytes were collected from human peripheral blood mononuclear cells (PBMCs) obtained from blood buffy coats according to the previously described protocol [29,30]. Blood sample (20 mL) from the veins of healthy volunteers was collected into sterile tubes containing EDTA. According to the manufacturer’s instructions, PBMCs were separated by gradient centrifugation on Ficoll–Hypaque (GE Healthcare, Wauwatosa, WI, USA) containing anti-CD14 microbeads (Miltenyi Biotec GmbH, Bergisch Gladbach, Germany). Cells were washed twice with phosphate buffered saline (PBS) and then resuspended in Roswell Park Memorial Institute (RPMI) 1640 medium (Mediatech, Manassas, VA, USA) containing 10% FBS, 100 μg/mL streptomycin, 100 U/mL penicillin, and 0.25 g/mL Amphotericin B. Cells were labeled with CD3-PE and CD14-FITC and then assessed for the purity of CD14^+^ cells by flow cytometry, which showed >95% purity. The purified human CD14^+^ cells were differentiated into macrophages by adding 10 ng/mL human granulocyte-macrophage colony-stimulating factor (GM-CSF) (PeproTech, Rocky Hill, NJ, USA) for 6 days at 37 °C in 5% CO_2_, and were prepared for subsequent experiments.

### 2.9. Statistical Analysis

The results are presented as the means ± standard deviations, with *n* indicating the number of experiments. Comparisons among groups were performed by 1-way ANOVA followed by post hoc test. The *p*-value < 0.05 was considered significant.

## 3. Results

### 3.1. TGII Does Not Induce Macrophage Cytotoxicity

**Figure 1:**
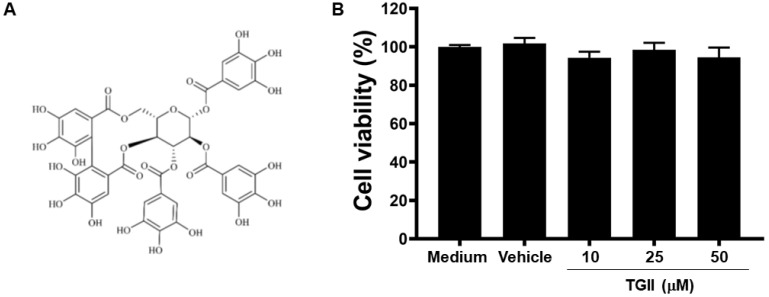
The effect of Tellimagrandin II treatment on the viability of RAW 264.7 cells. (**A**) Chemical structure of Tellimagrandin II (TGII). (**B**) RAW 264.7 cells were treated with medium, vehicle (0.1% DMSO), or TGII (10, 25, and 50 μM) for 24 h, and then measured for cell viability by Alamar Blue assay. Data are expressed as the means ± standard deviations (SD) of five independent experiments (*n* = 5).

Figure 1A illustrates the chemical structure of TGII. No significant differences were observed in cell viability between RAW264.7 cells treated with or without 50 μM TGII (Figure 1B), suggesting that TGII did not influence the cell viability in doses up to 50 μM. Consequently, all subsequent experiments were carried out using a maximal 50 μM dose of TGII. 

### 3.2. TGII Reduces LPS-Stimulated NO Production in Macrophages by Regulating NOS2 Transcription

**Figure 2:**
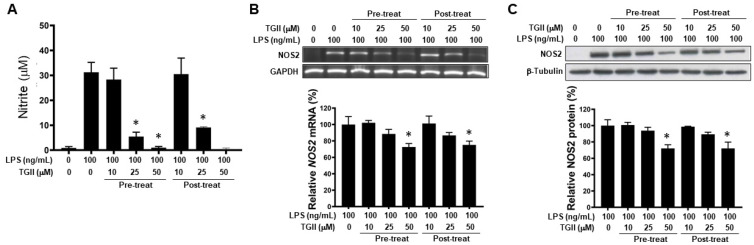
The effect of Tellimagrandin II on nitrite and NOS2 production in LPS-stimulated RA264.7 cells. RAW 264.7 cells were pre- or post-treated with the indicated concentrations of TGII. The nitrite (**A**), NOS2 mRNA (**B**) and protein (**C**) were quantified after 6 h, 3 h, and 6 h of LPS (100 ng/mL) stimulation, respectively. Bottom panels show the fold-changes compared to the control group (LPS only). * *p* < 0.05 vs. LPS-treated cells. Whole Western blot of (**C**) used for quantification is provided in Supplementary Figure S1.

LPS-induced NO production was significantly suppressed in RAW 264.7 cells which were pre-treated with 25 or 50 μM TGII 30 min prior to LPS stimulation compared with that in non-treated cells, in a dose-dependent manner (Figure 2A). Similar observations were also found in LPS-stimulated (for 30 min) RAW264.7 cells treated with 25 or 50 μM TGII, i.e., post-treatment (Figure 2A). Pre- or post-treatment with 50 μM TGII significantly attenuated LPS-induced NOS2 mRNA and protein expression in RAW 264.7 cells (Figure 2B,C).

### 3.3. TGII Suppressed LPS-Stimulated Macrophage Activation Is Associated with Increased Phosphorylated-p-38 Expression

**Figure 3:**
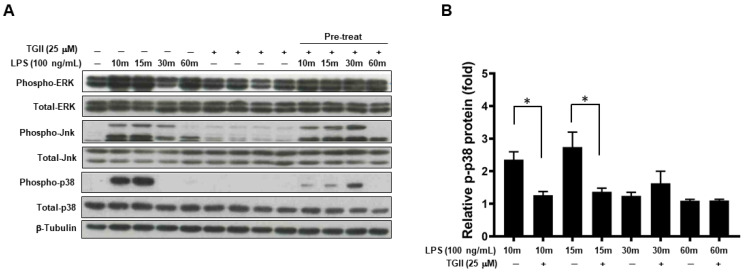
The effect of Tellimagrandin II on MAPKs expression in LPS-stimulated RAW264.7 cells. RAW264.7 cells were pre-treated with TGII (25 μM) for 30 min, and then stimulated with LPS (100 ng/mL) for 10 min, 15 min, 30 min, and 1 h. (**A**) After LPS treatment, phospho-Erk, phospho-Jnk and phospho-p38 were detected from total protein by Western blot (See Supplementary Materials). (**B**) The inhibition of p38 is presented as percentage change compared to that of the control group (LPS only). * *p* < 0.05. Whole Western blot of (**A**) used for quantification is provided in Supplementary Figure S2.

LPS-stimulated NO production is associated with MAPK kinase in a dose-dependent manner [31], thus, we evaluated whether TGII regulated LPS-stimulated NO production is through MAPK kinase. As shown in Figure 3, LPS-induced expression of phosphorylated p38 at 15 min was inhibited in RAW264.7 cells pre-treated with 25 μM TGII (*p* < 0.05). However, LPS-induced expression of phosphorylated Erk and Jnk at various time-points was not influenced in RAW264.7 cells pre-treated with 25 μM TGII. 

### 3.4. TGII Suppressed LPS-Stimulated Macrophage Activation Is Associated with the Increase in p-38 Downstream

**Figure 4:**
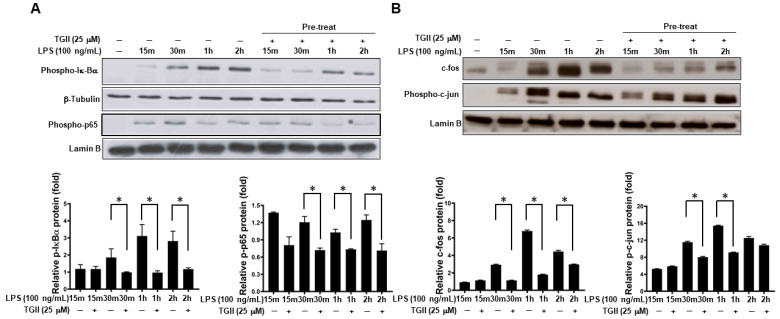
The effect of Tellimagrandin II on expression of NO-related transcription factors in LPS-stimulated RAW264.7 cells. RAW264.7 cells were pre-treated with TGII (25 mM) for 30 min, then stimulated with LPS (100 ng/mL) for 15 min, 30 min, 1 h, and 2 h. After LPS treatment, the cytosolic and nuclear extracts were isolated from RAW 264.7 cells. (**A**,**B**) The cytosolic extracts were analyzed for proteins of phospho-IκBα and β-tubulin by Western blot, while the nuclear extracts were analyzed for proteins of phospho-p65, c-fos, phospho-c-jun, and lamin B. Bottom panels show the percentage changes compared to those of the control group (LPS only). * *p* < 0.05. Whole Western blot of (**A**,**B**) used for quantification is provided in Supplementary Figures S3 and S4.

Downstream factors of p-38, including NF-κB, c-fos and c-jun, are involved in LPS-stimulated NO production in macrophages [31,32,33,34]. Thus, we determined whether TGII influences the expression of these proteins in LPS-treated RAW264.7 cells. For the NF-κB pathway, pre-treatment of 25 μM TGII for 30 min significantly repressed the expression of phospho-IκB-α and phospho-p65 in LPS-stimulated RAW264.7 cells compared to those without TGII treatment (Figure 4A). Similar observations were also found in the expression of c-fos and phospho-c-jun in LPS-stimulated RAW264.7 cells pre-treated with 25 μM TGII for 30 min (Figure 4B), although c-jun was not significantly different (data not shown).

### 3.5. TGII Inhibits COX-2 Signaling in LPS-Activated Macrophages

**Figure 5:**
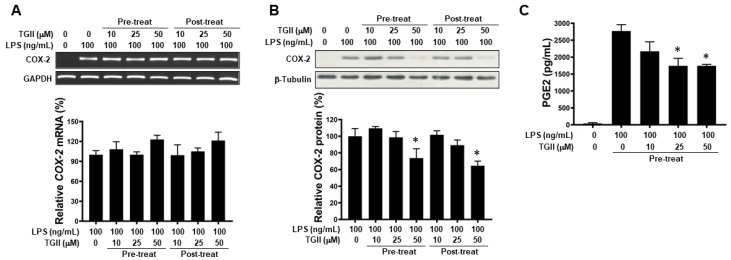
The effect of Tellimagrandin II on COX-2 expression and PGE2 production in LPS-stimulated RAW 264.7 cells. RAW 264.7 cells were pre- or post-treated with the indicated concentrations of TGII. The *COX-2* mRNA (**A**), COX-2 protein (**B**) and PGE2 production (**C**) were quantified after 3 h, 6 h, and 12 h of LPS (100 ng/mL) stimulation, respectively. Bottom panels show the percentage changes compared to those of the control group (LPS only). * *p* < 0.05. Whole Western blot of (**B**) used for quantification is provided in Supplementary Figure S5.

The anti-inflammatory effects of TGII on macrophages by regulating the COX-2 pathway were determined as shown in Figure 5A,B. Specifically, pre- or post-treatment with TGII did not attenuate LPS-induced *COX-2* gene expression, but decreased COX-2 protein in a dose-dependent manner. Subsequently, LPS-induced PGE2 overproduction was suppressed by TGII (Figure 5C).

### 3.6. Effects of TGII on PGE2 Production in CD14^+^ Monocyte-Derived Macrophages after LPS Stimulation

**Figure 6:**
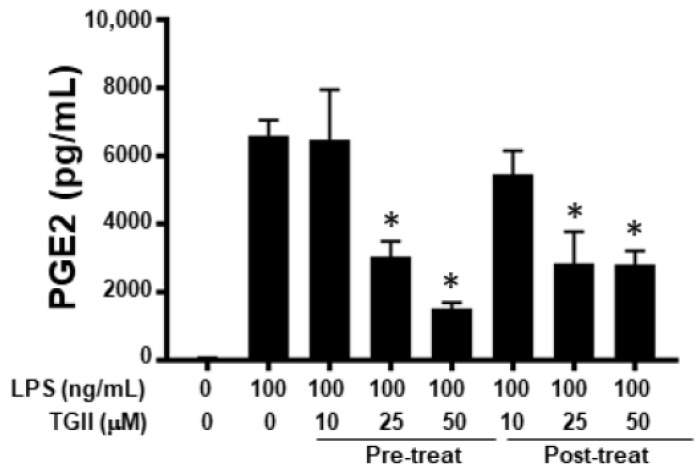
The effect of Tellimagrandin II on PGE2 production in LPS-stimulated human CD14^+^ monocyte-derived macrophages. The primary cells, human CD14^+^ monocyte-derived macrophages, were pre- or post-treated with the indicated concentrations of TGII for 30 min before LPS stimulation, or 30 min after LPS stimulation, and then PGE2 production was quantified after 24 h of LPS (100 ng/mL) stimulation, respectively. * *p* < 0.05, the concentrations compared to the LPS-stimulated group.

To confirm the anti-inflammatory activity of TGII in primary macrophages, we also used human CD14^+^ monocyte-derived macrophages to carry out ex vivo experiments. As shown in Figure 6, LPS-induced PGE2 production was inhibited by pre- or post-treatment of TGII. However, TGII-inhibited LPS-induced NO production in these cells was near the limit of detection (data not shown), just like what has been shown by us [35] and others [36]. 

## 4. Discussion

**Figure 7:**
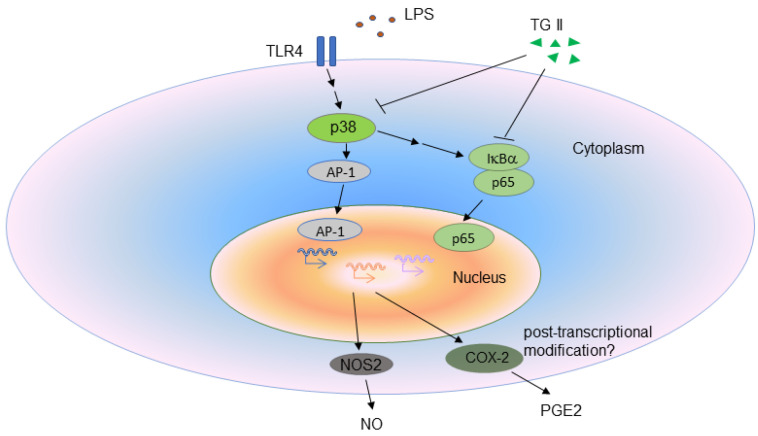
Proposed mechanism depicting the effect of Tellimagrandin II on LPS-stimulated macrophages.

In the present study, we show expanded evidence that TGII represses inflammatory activity on LPS-stimulated macrophages (Figure 7). The productions of NO and PGE2 were reduced with TGII treatment, in addition to both mRNA and protein of the NOS2, in a dose-dependent manner. Simultaneously, upstream factors of NOS2 and PGE2, including p38, NF-κB, c-fos and COX-2, were inhibited in LPS-stimulated macrophages with TGII treatment, suggesting that a possible therapeutic approach to inflammatory diseases such as sepsis may focus on anti-inflammatory agents to suppress the proinflammatory response.

A lot of therapeutic agents have been developed to treat sepsis, including antibodies against LPS, antitumor necrosis factor (TNF) agents, Toll-like receptor 4 (TLR4) antagonists, drugs targeting the coagulation cascade, and drugs targeting platelet-activating factor (PAF) [37,38]. In a clinical trial, drotrecogin alfa (trade name, Xigris), a recombinant human form of protein C, was found to block coagulation, inhibit inflammatory effects, and preserve organ function [39,40]. However, the therapeutic results for sepsis were unsuccessful owing to intolerable side effects, and the drug was withdrawn from the market in 2012 prior to Food and Drug Administration (FDA) approval for clinical use to treat sepsis. In the present study, no significant cytotoxic effects were noted for RAW 264.7 cells at TGII concentrations up to 50 μM, suggesting that TGII may have a larger “therapeutic range” for the treatment of inflammatory diseases such as sepsis. In particular, both pre- or post-treatment with TGII reduced NO production in LPS-stimulated macrophages, suggesting that TGII may have therapeutic effects for inflammatory diseases or the ability to prevent inflammatory diseases. The inhibition of pathophysiological NO has been suggested as a therapeutic target for sepsis [41]. 

Lipid mediators such as prostaglandins (PGs) have received widespread attention for their roles in mediating inflammatory and immune responses to severe infection. PG is an oxygenated metabolite of arachidonic acid, a small molecule, and it participates in various roles in regulating pathophysiological reactions in sepsis. The synthesis, catabolism, and signal transduction of PG have been studied as targets for the treatment of sepsis, especially when used in combination with antimicrobial agents and supportive care. Early studies of targeted PG synthesis in the treatment of sepsis involved the inhibition of cyclooxygenase (COX), which is the first important enzymatic step in the metabolism of arachidonic acid into biologically active PG [42,43,44]. For example, PGE2 synthesis and signal transduction are increasingly being studied as targets for immunotherapy in severe infections [45,46]. However, human pharmacological trials of COX inhibitors did not provide consistent beneficial findings [47]. The lack of effect in human studies has not been explained, but a possible explanation has been shown in many animal studies of sepsis; COX inhibitors are given before severe inflammation or infection occurs [48,49]. Interestingly, the present study demonstrated that both pre- and post-treatment with TGII significantly inhibited the protein levels of COX-2 in LPS-stimulated macrophages. Subsequently, downstream of COX-2, PGE2 was also suppressed in these cells. This suggests that TGII may also serve as a potential agent for the treatment of sepsis by regulating COX-2-mediated PGE2 production. In particular, TGII, with its inhibitory effects on COX-2, may also play a therapeutic role in the management of patients with SARS-CoV-2 because COX-2 inhibitors have been reported to be effective for treating patients with COVID-19 [50]. 

Polyphenols affect the inflammatory process by inhibiting pro-inflammatory cytokines (e.g., IL-1β, IL-6, IL-8, TNF-α and COX-2) involved in the metabolism of arachidonic acid; polyphenols also exhibit anti-inflammatory activity at many levels via NF-κB inhibition and NOS2, MAPK, and growth factor regulation [31,32,34,51]. Similarly, TGII provides anti-inflammatory activity by regulating the expression of these factors in LPS-stimulated macrophages. Interestingly, TGII significantly suppresses NOS2 expression at both the transcriptional and translational levels in LPS-stimulated RAW 264.7 cells, while it significantly suppresses COX-2 expression at the protein level in these cells.

The promoter of the NOS2 gene is responsible for excessive NO production by LPS in sepsis [52]. The activation of TLR4 by LPS leads to the phosphorylation of the NF-κB kinase (IKK) inhibitor, which phosphorylates the NF-κB inhibitor (IκB) and releases the transcription factor NF-κB [53]. Further, NF-κB translocates from the cytoplasm to the nucleus where it interacts with NF-κB elements in the 5′ flanking region of NOS2 to trigger NOS2 transcription, and TLR4 activated by LPS also induces AP-1-related transcription factors (c-fos and c-jun), leading to NOS2 transcription [53]. In the present study, TGII significantly inhibited these NOS2-related transcription factors in LSP-stimulated macrophages. Based on these findings, we suggest that TGII-repressed NOS2 expression may be mediated by regulating these transcription factors in LPS-stimulated macrophages. Certainly, it merits further investigation.

Unlike the anti-inflammatory activity observed for other polyphenol compounds (e.g., phlorofucofuroeckol A and sulfuretin) that have an effect on mRNA and protein expression of COX-2 in LPS-stimulated macrophages [31,32,51], both pre- and post-treatment of TGII only reduced COX-2 protein in LPS-stimulated macrophages. These results imply that the traditional polyphenols and COX-2 protein-selective TGII have different chemical properties, suggesting that TGII poses post-transcriptional modification effects on COX-2 protein.

## 5. Conclusions

TGII is an inhibitor of p-38 and reduces inflammatory response through its suppression of the production of NO and PGE2 in LPS-stimulated macrophages. These findings suggest that TGII may exert a novel therapeutic effect on sepsis and other inflammatory diseases by suppressing the release of NO and PGE2. Preliminary pharmacologic tests show that TGII may provide potent inhibitory effects on the NO and COX-2 pathways, exerting an anti-inflammatory effect through modulating the synthesis of several mediators involved in the inflammatory process. This appears to be the basic mechanism of anti-inflammatory activity, suggesting that TGII as a clinical agent may elicit a beneficial effect on inflammatory diseases such as sepsis, as well as other inflammatory diseases such as COVID-19, osteoarthritis, rheumatoid arthritis, spondylosis and slipped disk, although effects on these diseases were not investigated in the present study. Further study is needed to evaluate the pharmacologic mechanisms of TGII across a broad spectrum of inflammatory diseases.

## Data Availability

The data used to support the findings of this study are included within the article and its Supplementary Information Files.

## Supplementary Materials

The following are available online at https://www.mdpi.com/article/10.3390/life11050411/s1, Table S1: Primer design for the NOS2, COX-2 and GAPDH genes, Figure S1: Whole blot showing all the bands of Western blot in Figure 2C are summarized, Figure S2: Whole blot showing all the bands of Western blot in Figure 3A are summarized, Figure S3: Whole blot showing all the bands of Western blot in Figure 4A are summarized, Figure S4: Whole blot showing all the bands of Western blot in Figure 4B are summarized, Figure S5: Whole blot showing all the bands of Western blot in Figure 5B are summarized.
