# Supplementary material for: Lipopolysaccharide-Induced Nitric Oxide and Prostaglandin E2 Production Is Inhibited by Tellimagrandin II in Mouse and Human Macrophages
Source: Life (Basel). 2021 Apr 30;11(5):411. doi: 10.3390/life11050411 (PMC8146495; doi:10.3390/life11050411)

life

MDPI

Supplementary Materials

Lipopolysaccharide-Induced Nitric Oxide and Prostaglandin E2 Production Is Inhibited by Tellimagrandin II in Mouse and Human Macrophages

Table S1. Primer design for the NOS2, COX-2 and GAPDH genes.

Gene name	Primer	Sequences (5´-3´)
**NOS2**	Sense	CCCTTCCGAAGTTTCTGGCAGCAGC
	Antisense	GGCTGTCAGAGAGCCTCGTGGCTTTGG
**COX-2**	Sense	CATTGATGGTGGCTGTTTTG
	Antisense	GTTGCTGGGGGAAGAAATGT
**GAPDH**	Sense	TCCACCACCCTGTTGCTGTA
	Antisense	ACCACAGTCCATGCCATCAC

Figure S1. Whole blot showing all the bands of Western blot in Figure 2(**C**) are summarized.

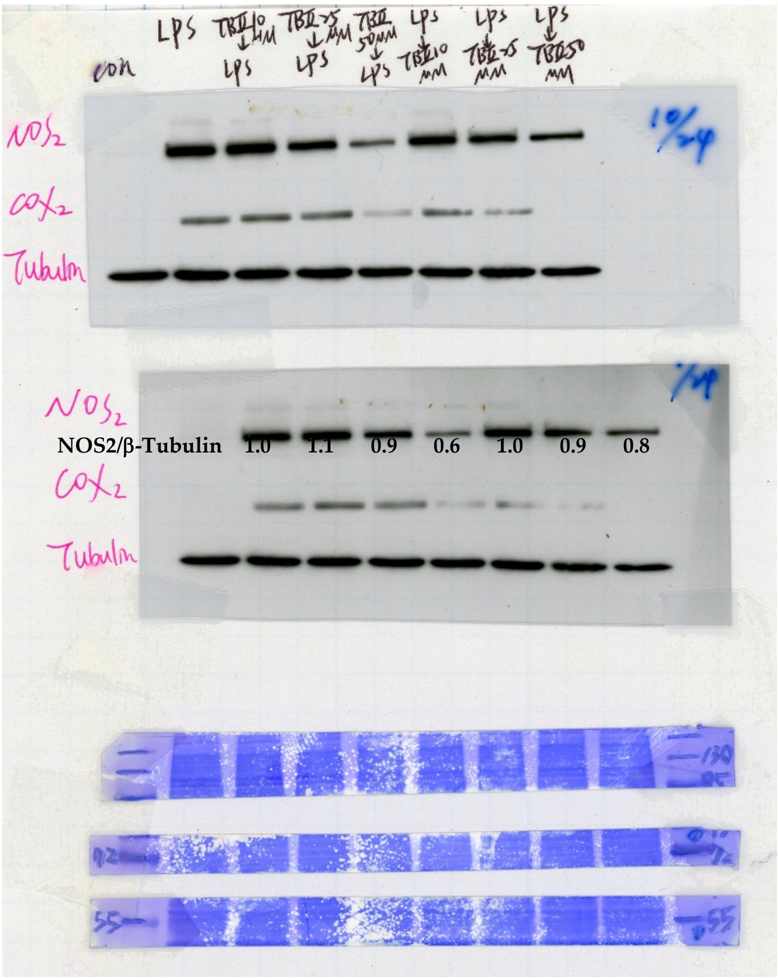

Figure S2. Whole blot showing all the bands of Western blot in Figure 3(**A**) are summarized.

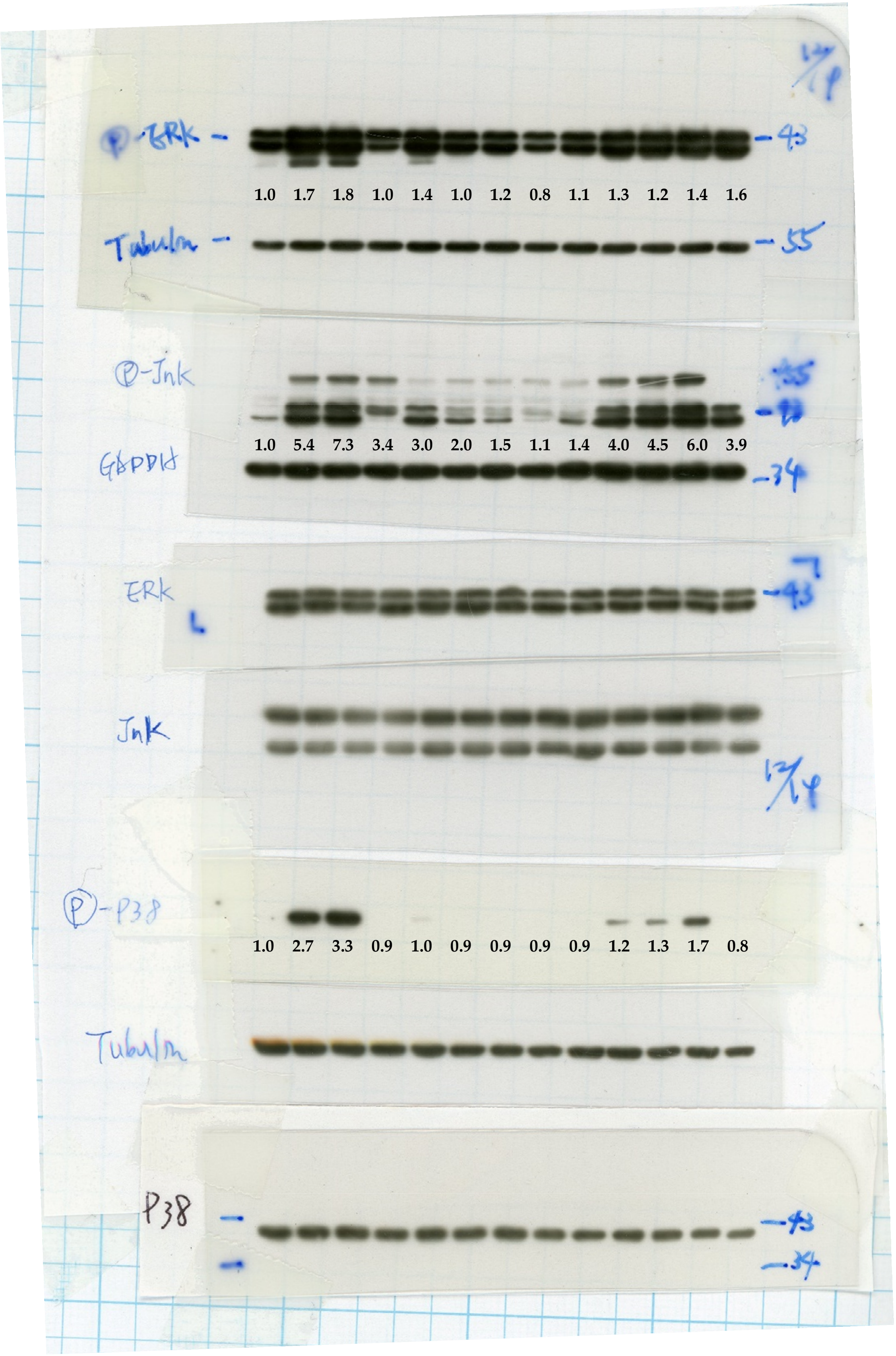

Figure S3. Whole blot showing all the bands of Western blot in Figure 4(**A**) are summarized.

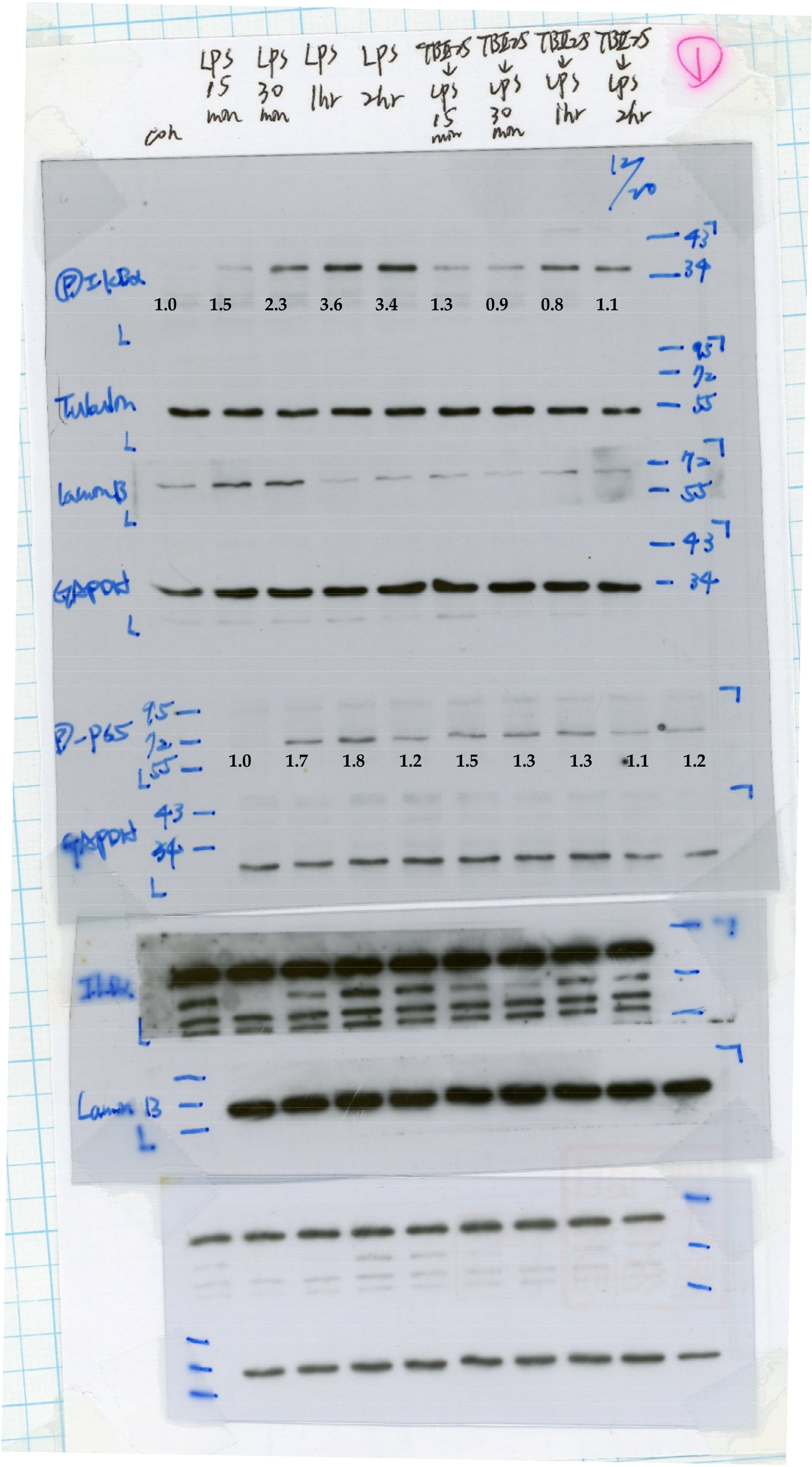

Figure S4. Whole blot showing all the bands of Western blot in Figure 4(**B**) are summarized.

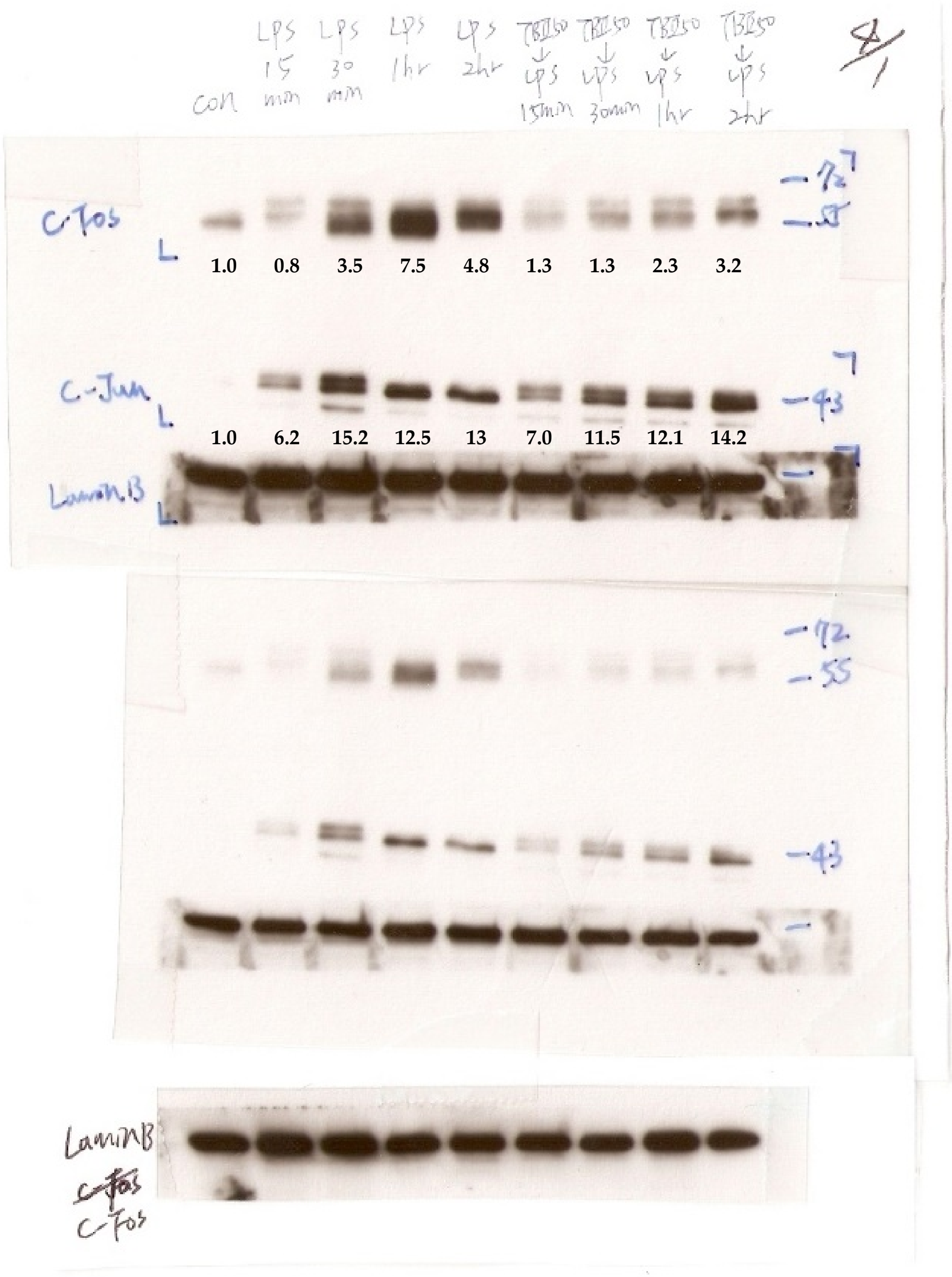

Figure S5. Whole blot showing all the bands of Western blot in Figure 5(**B**) are summarized.

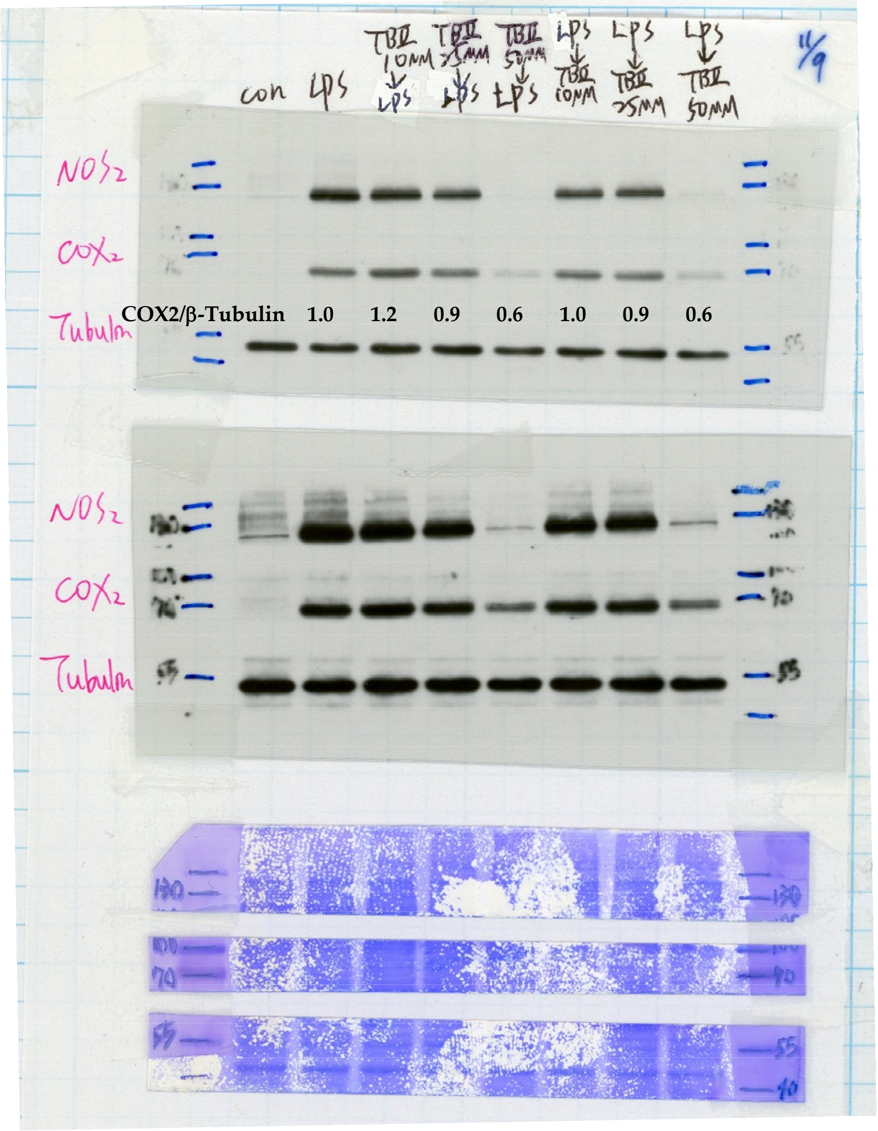

Supplement: Supplementary file 1 [file life-11-00411-s001.zip › life-1164178-supplementary.pdf]
